# The Provocative Roles of Platelets in Liver Disease and Cancer

**DOI:** 10.3389/fonc.2021.643815

**Published:** 2021-07-21

**Authors:** Preeti Kanikarla Marie, Natalie W. Fowlkes, Vahid Afshar-Kharghan, Stephanie L. Martch, Alexey Sorokin, John Paul Shen, Van K. Morris, Arvind Dasari, Nancy You, Anil K. Sood, Michael J. Overman, Scott Kopetz, David George Menter

**Affiliations:** ^1^ Department of Gastrointestinal Medical Oncology, The University of Texas MD Anderson Cancer Center, Houston, TX, United States; ^2^ Department of Veterinary Medicine and Surgery, The University of Texas MD Anderson Cancer Center, Houston, TX, United States; ^3^ Division of Internal Medicine, Benign Hematology, The University of Texas MD Anderson Cancer Center, Houston, TX, United States; ^4^ Department of Surgical Oncology, The University of Texas MD Anderson Cancer Center, Houston, TX, United States; ^5^ Department of Gynecologic Oncology and Reproductive Medicine, The University of Texas MD Anderson Cancer Center, Houston, TX, United States

**Keywords:** platelets, minimal residual disease, metastasis, wounding, repair, regeneration, first responders

## Abstract

Both platelets and the liver play important roles in the processes of coagulation and innate immunity. Platelet responses at the site of an injury are rapid; their immediate activation and structural changes minimize the loss of blood. The majority of coagulation proteins are produced by the liver—a multifunctional organ that also plays a critical role in many processes: removal of toxins and metabolism of fats, proteins, carbohydrates, and drugs. Chronic inflammation, trauma, or other causes of irreversible damage to the liver can dysregulate these pathways leading to organ and systemic abnormalities. In some cases, platelet-to-lymphocyte ratios can also be a predictor of disease outcome. An example is cirrhosis, which increases the risk of bleeding and prothrombotic events followed by activation of platelets. Along with a triggered coagulation cascade, the platelets increase the risk of pro-thrombotic events and contribute to cancer progression and metastasis. This progression and the resulting tissue destruction is physiologically comparable to a persistent, chronic wound. Various cancers, including colorectal cancer, have been associated with increased thrombocytosis, platelet activation, platelet-storage granule release, and thrombosis; anti-platelet agents can reduce cancer risk and progression. However, in cancer patients with pre-existing liver disease who are undergoing chemotherapy, the risk of thrombotic events becomes challenging to manage due to their inherent risk for bleeding. Chemotherapy, also known to induce damage to the liver, further increases the frequency of thrombotic events. Depending on individual patient risks, these factors acting together can disrupt the fragile balance between pro- and anti-coagulant processes, heightening liver thrombogenesis, and possibly providing a niche for circulating tumor cells to adhere to—thus promoting both liver metastasis and cancer-cell survival following treatment (that is, with minimal residual disease in the liver).

## Hemostasis, Chronic Trauma, and Cancer

Trauma triggers clotting mechanisms that prevent excessive blood loss from organs and the body in general. The initial hemodynamic response to trauma is contractions in vessel walls of smooth muscle resulting in vasoconstriction to limit blood loss—a response that is highly transient. Fortunately, vasoconstriction of damaged blood vessels is concomitant with the initiation of the coagulation process. Indeed, trauma also induces exposure of platelets to prothrombogenic extracellular matrix and tissue factor (i.e. platelet tissue factor, tissue thromboplastin, factor III, or CD142). The associated mechanisms of clot formation in primary hemostasis include platelet activation, adhesion, and aggregation resulting in platelet micro-plug formation. Secondary homeostasis includes strengthening of the expanding plug—a process that involves clotting factors—and formation of fibrin (1). Wound healing, like cancer, involves the invasion of immune cells, fibroblasts, and other stromal cells to cleanse and repair the lesion (2–4). During the acute phase of wound healing, the subsequent repair and resolution involves the additional action of anticoagulant proteins to break down the fibrin mesh. Once sufficient healing of the wound has occurred, a clot dissolution process takes place with the activation of plasminogen. In the case of chronic inflammation associated with non-alcoholic steatohepatitis (NASH), other liver diseases (5), or cancer, the resolution phase stalls or is suppressed by the non-healing abnormal tissue (2–4). Each organ has unique endogenous cellular characteristics and tissue matrices that govern interactions of coagulation and immune responses. The liver contains a unique microenvironment with a heterogeneous mix of many cell types (6). This unique cellular complexity provides distinctive biological influences on tissue wounding and repair (7). While the intricacies of this unique liver biology and its interface with platelets is not well understood, platelets do help in the infiltration of immune cells by initiating the tumor immune response (8).

## Platelets as First Responders

Platelets can be considered first responders in the context of wounding, tissue repair, and inflammatory and immune responses that occur with cancer progression and metastasis. Platelets are important anucleate elements of the immune and hemostatic systems. Their genesis occurs when membrane-bound organelles containing cytoplasmic extrusion are released as small (2-3 μm) blebs into the blood stream. This blebbing, subcellular, genesis process occurs from surfaces of megakaryocytes, the largest cells in the body, and occurs primarily in the bone marrow. Normal human platelets are principally considered to be responsible for coagulation and fibrinolysis, but emerging data suggest a greater impact on immunology and cancer biology.

Resting platelets are plate-like discs that maximize planar surface interactions (9–11) that are biophysically concentrated toward the outer fluid-shear fields of flowing blood—much like silt movements associated with wave action (12–19). The liver sinusoids exhibit their own unique uneven wall/fluid-shear characteristics due to the open irregular sinusoidal vasculature compared to the typical vessels that are fully enveloped by a smooth continuous layer of endothelial cells and are subject to more uniform laminar field shear (20–23). These liver-sinusoidal vessel properties can change with liver fibrosis, metastasis, and damage from chemotherapy (24–28). Taken together, these properties enhance encounters with and recognition of any vascular wall lesions, wounds, or tumors. Should platelets encounter basement membrane or underlying matrix, they undergo receptor-mediated activation (29–34) connected to very rapid cytoskeleton- and membrane changes to form filopodia leading to adhesion (35–42). This rapid process occurs within seconds along with shape change and exocytic degranulation. In turn, degranulation encompasses the release of proteins, growth factors, cytokines, and lysozymes accompanied by a variety of bioactive lipids, small molecules, and other factors. Successful sequential recruitment of additional platelets and immune cells coupled with thrombogenesis ultimately seals any tissue gaps and initiates recruitment of other immune cells.

### Platelet-Derived Serum Contents

The primary serum constituents resulting from platelet-initiated coagulation, growth, and wound repair are derived from intracellular sources (43–47). Circulating, resting platelets contain multiple storage granules: 1) alpha-granules (α-granules) 2) dense granules (δ-granules) 3) lysosomes, and 4) microparticles (48–50). There is also a newly described T-granule that primarily contains toll-like receptors 9 (51).

### Platelet α-Granules

Of the various types of platelet granules, the most common are α-granules, which constitute 10% of the platelet volume and number 50-80 per platelet (52). Two pathways contribute to α-granule formation. De novo synthesis occurs when the trans-Golgi network generates a core structure that attracts clathrin to form a lattice structure and interacts with coat proteins like adaptor-protein 1 resulting in clathrin-coated pits. After invagination, these pits bud forming membrane-bound vesicles that end up in early endosomes. A strictly endocytic vesicle pathway also exists involving adaptor-protein 2 that is involved in forming subsets of endosomes. Alpha-granule subset lineages mature in multivesicular bodies through the engagement of vacuolar protein sorting-associated protein (-33B and -16B) that are involved in sorting certain cargo from the trans-Golgi network. Neurobeachin-like protein 2 is another molecule that helps drive α-granule development and secretion. Mature α-granules are released *via* exocytosis following platelet stimulation *via* a process involving dynamin-related protein-1 and other cytoskeletal elements (53–55).

#### α-Granule Adhesion and Coagulation Molecules

Adhesion proteins released from α-granules help to stimulate the rapid arrest of platelets in circulation mediated by vonWillebrand factor (vWF), fibrinogen, integrins (αIIbβ3 and αvβ3), P–selectin, thrombospondin, and fibronectin. These proteins regulate interactions between platelets and endothelial cells, exposed basement membrane extracellular matrix, leukocytes, neutrophils monocytes, tumor cells, and other platelets. The same α-granules also release prothrombin, fibrinogen, factor V, and factor VIII that stimulate and promote coagulation and fibrin formation (56–66).

#### α-Granule Growth and Angiogenesis Factors

Infiltrating- and tissue-resident immune cells, stromal cells, and fibroblasts are stimulated by α-granule factors. Growth factors include platelet-derived growth factor (PDGF), basic fibroblast growth factor (48, 67), epidermal growth factor (EGF) (68), hepatocyte growth factor (69, 70), insulin-like growth factor 1 (71, 72), and transforming growth factor-beta (TGFβ) (73–79)—all of which can induce cell-type- and function-specific proliferation and immunomodulation. Pericytes, smooth muscle fibroblasts (myofibroblasts), and endothelial cells are stimulated by α-granule-released pro-angiogenic and anti-angiogenic factors that include vascular endothelial growth factors (VEGF)-A and -C) (80, 81), angiopoietin-1, angiostatin, and sphingosine-1-phosphate (48, 59, 67, 80, 82–85).

#### Platelet-Dense δ-Granules

Dense-granules are derived from the platelet endosomal lineage compartment. Early endosomes are currently thought to mature in multivesicular bodies. Like melanosomes, biogenesis of lysosome-related organelles complex (BLOC1 and -2) are involved with cellular exit of tubular structures that transport cargo from the endosomes to maturing δ-granules (83). The adapter-protein complex-3 may also elicit biogenesis of lysosome-related organelle complexes potentially involving BLOC1 and BLOC2. δ-granules that are generated from BLOC-containing organelle complexes can number 3-8 per human platelet and primarily contain bioactive small molecules. These δ-granules are released into the bloodstream during degranulation to enhance platelet activation, adhesion, and stabilization at sites of vascular damage. A number of ions (including calcium, magnesium, phosphate, and pyrophosphate) are released into the developing lesion microenvironment and influence platelet aggregation, clot progression, and wound evolution. Platelet δ-granules also actively accumulate and sequester nucleotides ATP, GTP, ADP, GDP and cyclic nucleotides *via* multidrug-resistance protein 4 (i.e., ABCC4), a transport pump for cyclic nucleotides and nucleotide analogs (86, 87). The exposure of tetraspanins and lysosomal-associated membrane protein-2 in association with platelet activation also occurs during release of δ-granules (88–91). Platelet δ-granules also release transmitters like serotonin (5-HT), epinephrine, and histamine (92, 93), which influence vascular function, macrophages, thrombosis, liver regeneration, and cancer progression (70, 94–97). Both α- and δ-granule release amplify secondary platelet responses, initiate wound repair, and influence cancer-cell proliferation (55, 98–100).

#### Granule Release as Essential Components of the Immune System

Although platelets are principally considered to be responsible for coagulation and fibrinolysis, data suggest that platelets also serve as key effectors in immunological responses. Platelet function in normal wound biology contributes to potential pathogen clearance, tissue repair, and resolution of inflammation. Once a clot is formed, pro-inflammatory mediators attract immune cells to sterilize the resulting wound. These factors include C-X-C motif chemokines such as CXCL1 (GRO-α), CXCL4 (PF4), CXCL5 (ENA-78), CXCL7 (PBP, β-TG, CTAP-III, NAP-2), CXCL8 (IL-8), CXCL12, and stromal-cell-derived factor-α (101). Platelet-derived CXCL12 is involved in mediating inflammation, immune response resolution, and repair mechanisms within sites of tissue injury (102). CXCL12 also binds CXCR4 and CXCR7 and regulates monocyte/macrophage functions (103). CXCL4 and CXCL7 are the most abundant α-granule proteins and—following CXCL12 binding along with chemokine C-X-C-motif ligand 11 (CXCL11) and macrophage migration-inhibitory factor—help to dynamically modulate wound site biology (104, 105). In one study, when platelets were co-cultured with monocytes, they predominantly differentiated into CD163+ macrophages (103, 106), which may involve EP4-receptor stimulation (107). In cardiovascular disease, CD163+ elevation elicits the differentiation of a distinct, alternative, non–foam-cell anti-inflammatory macrophage that, in turn, promotes angiogenesis, leakiness, inflammation, and plaque progression *via* the CD163/HIF1α/VEGF-A pathway (108). CD163 has been used as a biomarker of the anti-inflammatory M2 macrophage phenotype in tumor-associated macrophages and has been associated with tumor progression in a number of cancers including colorectal cancer (CRC) (109). Platelet α-granules also release additional C-C motif chemokines that include: CCL2 (MCP-1), CCL3 (MIP-1α), and CCL5 (RANTES), CCL7 (MCP-3); and CCL17 (TARC) along with IL1-β, PAF acetyl hydrolase, and LPA (83). Other immuno-active molecules include C1 inhibitor and immunoglobulin-G; while other hemostasis-related α-granule proteins include albumin, α1–antitrypsin, high-molecular-weight kininogen and Gas6 (92, 93).

Degranulation and surface interactions of platelets with immune cells induce biological responses by leucocytes and progenitor- and endothelial cells at the site of pathogen permeation or vascular injury inflow. Platelet interactions with neutrophils, monocytes, and lymphocytes activate and promote platelet-leukocyte aggregates that immobilize and eliminate pathogens from spreading further. Platelets can also phagocytize pathogens directly. Platelet toll-like receptors (TLRs) also recognize and respond to pathogens in the gut microbiome (110–112).

### Platelets in Wound Resolution

Platelets release significant amounts of factors from α-granules such as α2-macroglobulin, plasminogen activator, plasminogen, and plasminogen-activator inhibitor type-1 that help with fibrinolysis, clot dissolution, inflammation resolution, and wound repair (113–117). They also release tissue remodeling enzymes involved in wound repair that include matrix metalloproteinases (MMP-1, MMP-2, MMP-3, MMP-9, MT1-MMP, MMP-14), tissue inhibitor of metalloproteinases (TIMPs; TIMP-1, TIMP-2 and TIMP-4), as well as a disintegrin and metalloproteinases (ADAMs; ADAM-10, ADAM-17, ADAMTS-13) (118, 119). Platelet-activating factor induces the expression of a number of tissue-remodeling proteases (120–122). PDGF also stimulates tissue remodeling hepatic stellate cell (HSC) proliferation and fibrosis in the liver that involves tissue-remodeling proteases (123). MMPs secreted by activated platelets play an integral role in tumor spread and the metastatic cascade by contributing in tissue remodeling and stimulating tumor-cell transmigration and invasion of surrounding tissues, blood vessels, and liver sinusoids (124–128).

### Platelet Lysosomes

Lysosome function in platelet biology and hemostasis is not well characterized (50, 55, 90, 91, 98, 129–134). Because they release phospholipase A, protease, and glycohydrolase enzymes, this suggests that lysosomes play a role in platelet responses and dissolution of clots (129, 132–134). Successive waves of platelet activation, adhesion, aggregation, and stabilization allow these first responders to activate specific subsets of cytoskeletal changes to recognize and secure vascular lesions.

### Platelet Clearance

The body is thought to produce and clear 10^11^ platelets per day (135, 136); clearance occurs primarily in the liver and spleen. Clearance mechanisms include senescence and apoptosis driven by Bcl-xL and the proapoptotic molecules Bax and Bak, which set the internal clock for platelet lifespan in conjunction with BH3-only proteins, mitochondrial permeabilization, phosphatidylserine exposure, and lectin-mediated recognition of platelet glycans (135–137). The vWF or antibodies binding to platelet surface glycoproteins under fluid shear induce mechanosensory signaling that leads to ADAM17 and phosphatidylserine exposure along with desialylation (138). These molecular changes are also commonly associated with platelet aging-related clearance and thrombocytopenia found in type 2B von Willebrand disease (139). Recognition of platelet glycans by the Ashwell-Morell receptor leads to clearance of senescent platelets by hepatocytes, macrophages, and other resident liver- or spleen immune cells. The complexities and temporal state of the platelet life-span clock and surface recognition by resident liver-immune cells is likely to influence the dynamics of liver diseases by selectively eliminating aging platelets and allowing fully functional platelets to exert their normal biological function.

### Platelet-to-Lymphocyte Ratios

Elevations in circulating platelet-to-lymphocyte ratios are often associated with inflammation and poor outcomes along with being linked to infections, inflammatory diseases, liver disease and cancer (140–146). The predictive value indicative of poor outcomes linked to elevated platelet-to-lymphocyte ratios has shown clear associations with liver transplantation, particularly when performed for hepatocellular carcinoma and liver metastasis (147–152).

Evidence exists in support of the predictive value of platelet-to-lymphocyte ratios as a general biomarker of inflammation but prospective case-control studies in each disease will help to further establish this hypothesis.

## Liver Cell and Organ Biology

**Figure 1:**
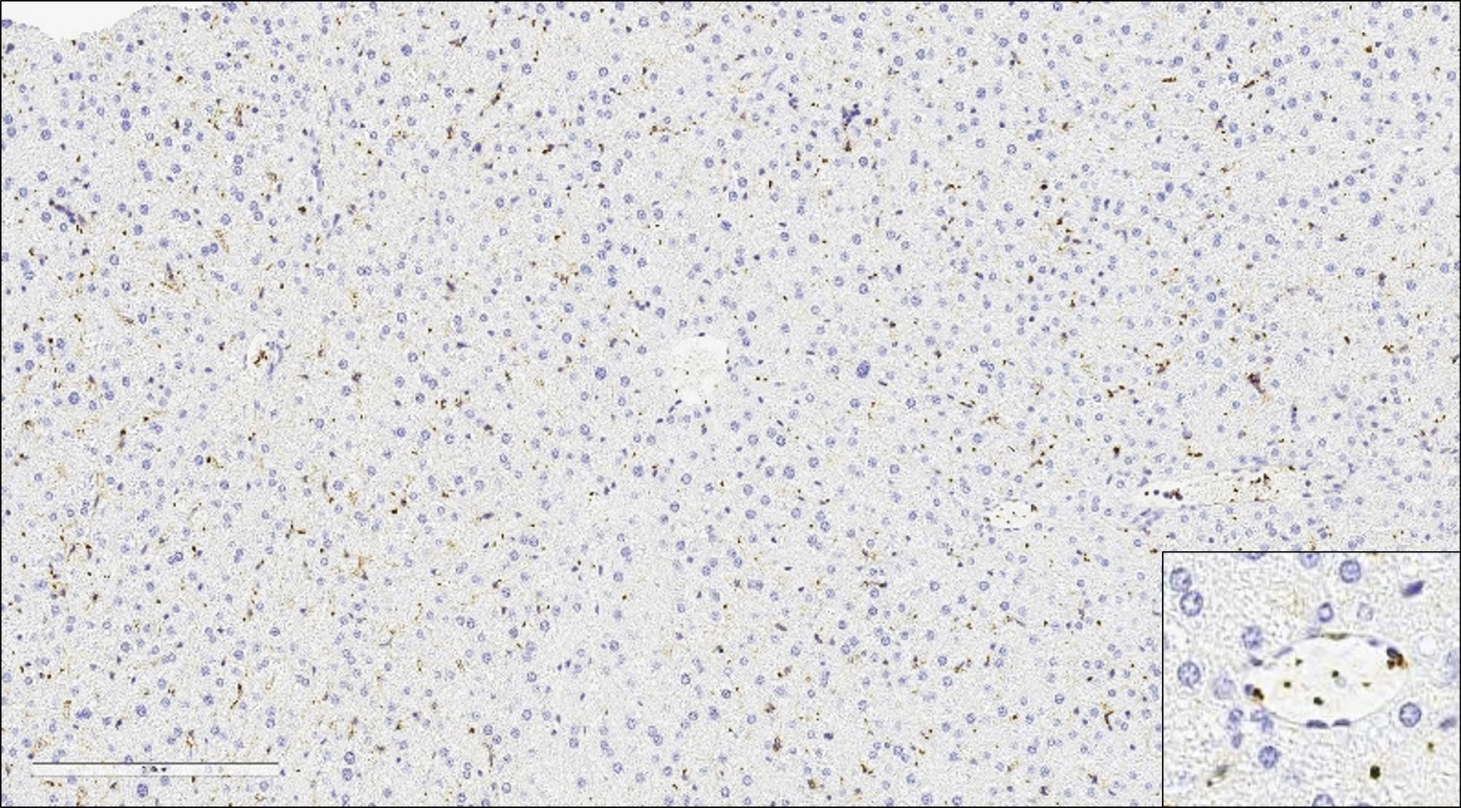
Platelets in Hepatic Sinusoids and Vessels in the Normal Liver. Large numbers of platelets immunoreactive for CD61 in the hepatic sinusoids and vessels of the normal murine liver (20x).

**Figure 2:**
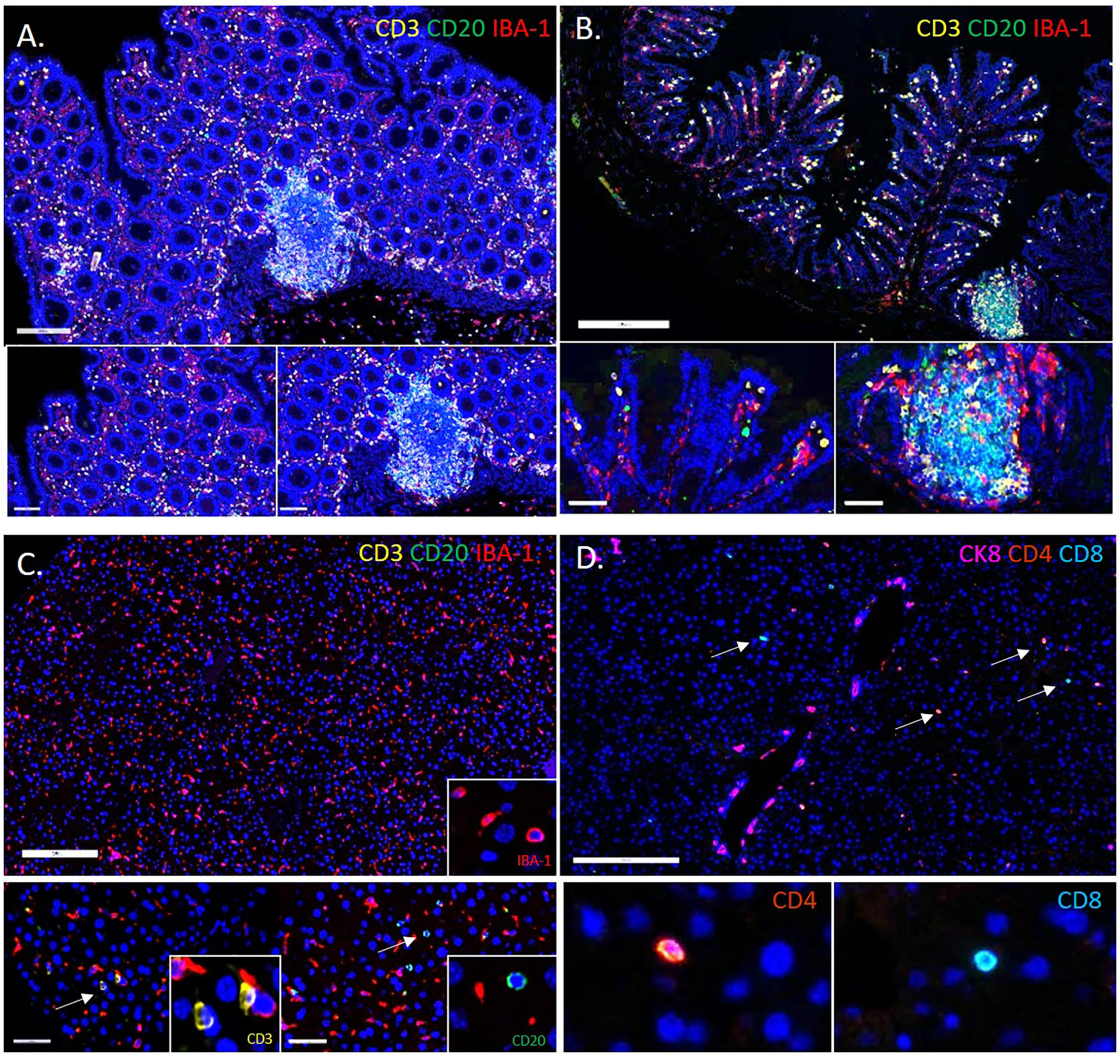
Comparison of the Immune Composition of the Microenvironment in the Colon and Liver. Multiplex immunofluorescent images showing the immune composition of the human **(A)** and murine **(B)** colon (20x). Large numbers of IBA-1+ macrophages (red), CD3+ T cells (gold), and CD20+ B cells (green). Immune cells reside in the lamina propria of the mucosa and Peyer’s patches. In comparison, the microenvironment of a healthy liver has far fewer CD3+ T cells (gold) and CD20+ B cells (green), and is instead predominated by specialized macrophages termed Kupffer cells (red) **(C)**. Insets show digitally magnified IBA-1+ macrophages (red), CD3+ T cells (gold) and CD20+ B cells (green) (not to scale). Image panel **(D)** shows both CD4+(orange) and CD8+(teal) T cells in the liver (insets are not to scale).

The normal liver exhibits unique heterogeneous and multifunctional organ properties, particularly at the cellular level (**Figures 1** **–** **5A**). Normal liver lobes are formed by parenchymal hepatocytes and non-parenchymal cells. Hepatocytes occupy nearly 80% of the total liver volume, play a role in innate immunity, and are responsible for much of the liver’s homeostatic and hemostatic functions using platelets widely distributed throughout the liver (**Figures 1** **–** **4**) (155, 156). Non-parenchymal cells represent 6.5% of the other liver-cell types that are organized around sinusoids.(**Figure 4**) The hepatic sinusoid walls are lined by sinusoidal endothelial cells (SECs), Kupffer cells, and HSCs along with intrahepatic lymphocytes, pit cells, and liver-specific, natural-killer cells (**Figures 2** **–** **4**) (157–167). Liver fibroblasts and myofibroblasts are thought to arise from multiple sources (168). Cholangiocytes and cells of the bile-canalicular system tend to associate preferentially with the hepatic arterial system (169).

**Figure 3:**
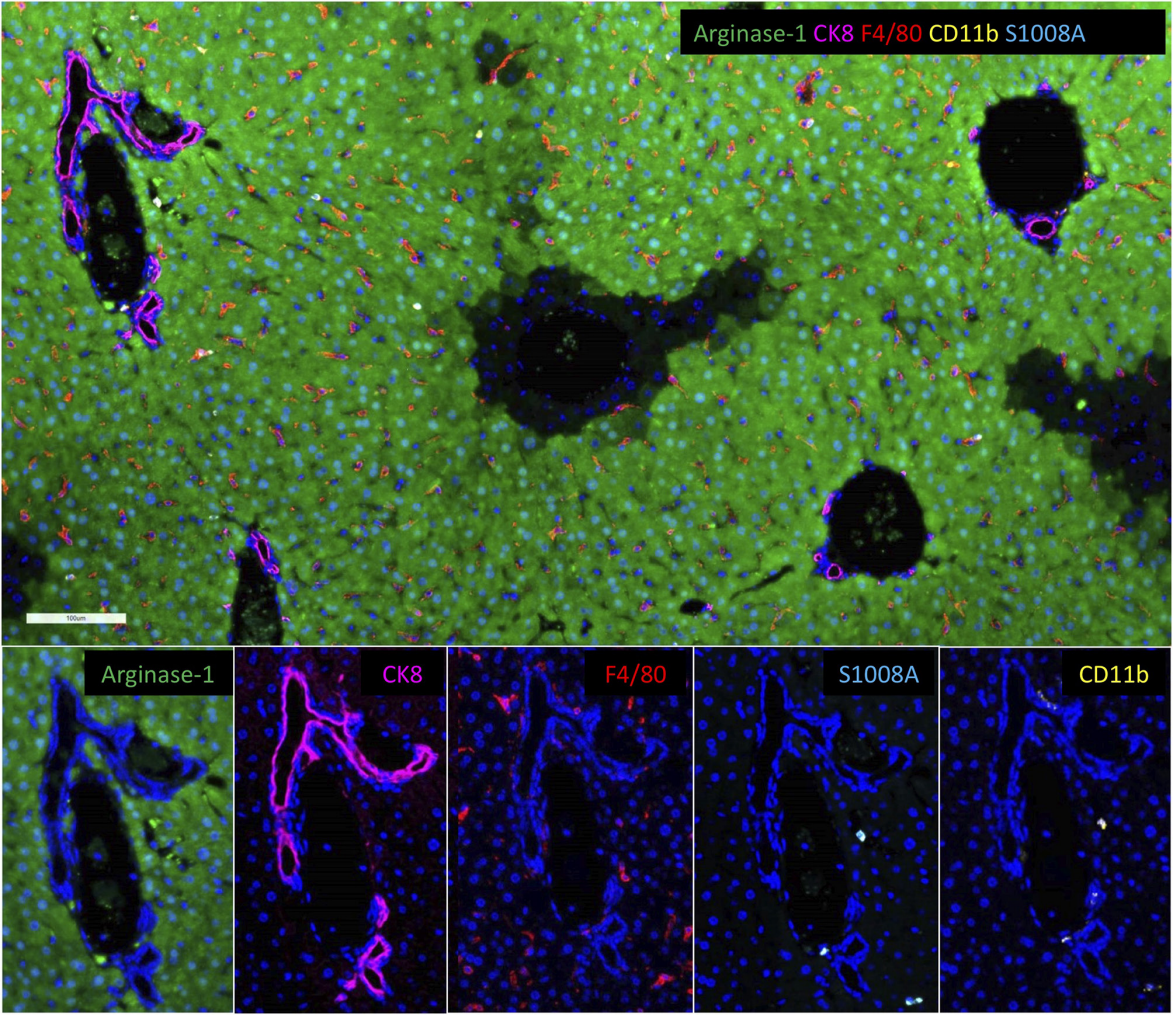
Resident and Bone Marrow-Derived Macrophages in the Liver. Multiplex immunofluorescent images of a normal murine liver showing the predominance of resident F4/80+ macrophages (red) with fewer bone marrow-derived CD11b+ macrophages (gold). Images also show S1008A expression (teal) by bone marrow-derived macrophages, but not resident macrophages. S1008A is involved in modulation of the immune response through cytokine secretion recruitment of leukocytes. Resident F4/80 macrophages also express arginase-1 consistent with M2, anti-inflammatory phenotype. Arginase-1 is widely expressed by hepatocytes.

**Figure 4:**
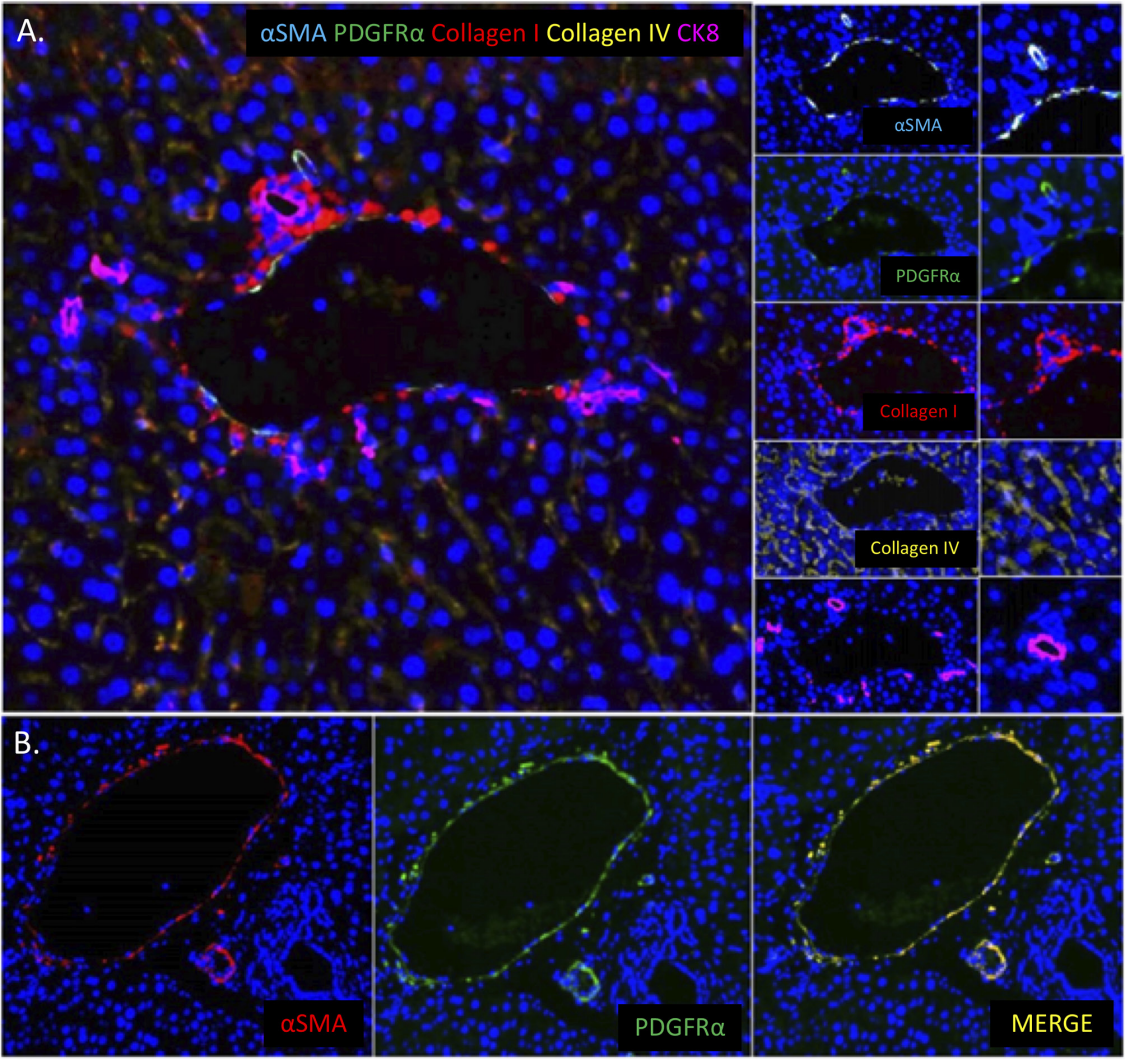
Stromal Composition of the Liver and PDGRα Expression by Hepatic Portal Vessels. **(A)** Multiplex immunofluorescent images of a normal murine liver showing the predominance of collagen type I (red) around portal areas and collagen type IV (gold) lining hepatic sinusoids. **(B)** Image showing the co-localization of PDGFRα (green) with α-smooth muscle actin positive portal veins and hepatic arterioles (red) in the portal area of the liver.

**Figure 5:**
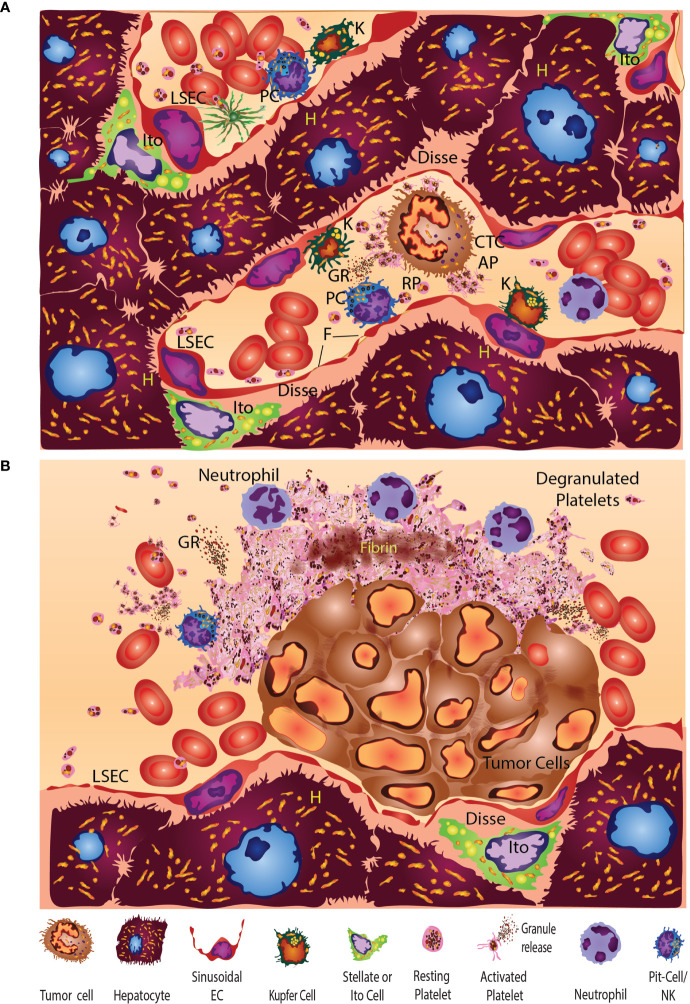
**(A)** The normal liver sinusoid exhibits unique heterogeneous multifunctional cells. Hepatocytes (yellow, H) occupy the bulk of the liver volume. Non-parenchymal liver cells represented are primarily organized around sinusoids. Liver sinusoidal endothelial cells (LSEC) line the walls of the sinus and have open-pore capillaries with 100-300 nm fenestrations (F). Resting Ito cells contain lipocytes or vitamin A and fat-storing vesicles (yellow droplets) are hepatic stellate cells (HSC). Resident immune cells consist of Kupfer cells (K), intrahepatic lymphocytes (IHL), pit cells (PC), or liver-specific natural-killer (LSNK) cells. Liver fibroblasts and myofibroblasts are thought to arise from multiple mesenchymal sources. Cholangiocytes and cells of the bile-canalicular system tend to associate preferentially ductal junctions that converge at the canals of Hering. The space of Disse surrounds the sinusoids and constitutes a stem-cell niche that harbors HSCs or liver-resident mesenchymal stem cells (MSCs) that patrol and regulate space function. HSCs or antler stem cells (ASC), MSCs and other cells freely migrate within the space of Disse and contribute to regeneration, liver fibrosis, carcinogenesis, and metastasis. HSC in the perisinusoidal space of Disse are characterized by the presence of well-branched cytoplasmic processes which contact endothelial cells. Circulating tumor cells (CTC) can stimulate resting platelets (RP) to become activated (AP) and release their stored granule contents. **(B)** Multiple studies (first reported by Dingemans) have shown that platelets respond within minutes of encountering platelets in the liver sinusoids (153, 154). Five minutes after injection, tumor cells had formed large emboli that were present in portal branches. Tumor emboli (TC) adhered to the vascular-wall liver sinusoidal endothelial cells (LSEC) without fully occluding the lumen. On the vascular-lumen side of the emboli, aggregated and degranulated platelets were common with leukocyte and neutrophil associations whereas erythrocytes were found near the non-platelet-involved tumor-cell surfaces. Degranulated platelets an outer zone of the platelet aggregates with a closely packed, activated-platelet inner zone containing fibrin deposits. Although not shown, in other studies Kupffer cells were engaged and engulfed by tumor cells.

Liver sinusoids, by contrast, are lined by discontinuous liver sinusoidal endothelial cells (LSECs) open-pore capillaries with 50-150 nm gaps on average that can also occur less frequently as 200-300 nm gaps as measured by SEM and atomic force microscopy (166, 167, 170, 171). These low-pressure sinusoids provide a porous separation from the underlying parenchyma, and thus play an essential role in maintaining metabolic and immune homeostasis while they actively contribute to disease pathophysiology (172). LSECs exhibit endocytic and scavenging functions and facilitate nutrient exchange, which contributes to receptor-mediated clearance of waste products *via* scavenger receptors (SR-B1, SCARF1) and immunoglobulin-G complexes (CD32b). Capillarization and dysfunction precede fibrogenesis. LSECs, along with other microenvironmental components, play an active role in liver disease (173). LSECs stretch receptors in particular can sense deformation and wall shear that leads to CXCL1 release *via* integrin-dependent activation of transcription factors regulated by Notch proteins and their interaction with the mechano-sensitive Piezo calcium channel (174). This results in the recruitment of neutrophils, and the generation of sinusoidal microthombi that can promote portal hypertension (174). Platelet-LSEC interactions may involve P-selectin expression (175). These platelet-LSEC interactions can also promote immune tolerance and recruit distinct immunosuppressive leukocyte subsets that allow persistence of chronic viral infections and facilitate tumor development (173). LSECs are also thought to modulate the tumor microenvironment and promote development of a pre-metastatic niche, which can drive formation of secondary liver tumors (176).

Liver sinusoids receive blood from the hepatic artery and portal vein that empty into central veins. The space of Disse surrounds the sinusoids and constitutes a stem-cell niche that harbors HSCs or liver-resident mesenchymal stem cells that patrol and regulate cellular function within this microenvironment (177–181). HSCs, antler stem cells, mesenchymal stem cells, and other cells freely migrate within the space of Disse and contribute to regeneration, liver fibrosis, carcinogenesis, and metastasis (177–185). The complexities of this resident stem-cell space may contribute to the liver becoming an organ of extra-medullary hematopoiesis (**Figure 5A**) that is also prone to tumor metastasis.

Among the many mediators involved in the intercellular communication in the liver, some include prostanoids, nitric oxide, endothelin-1, TNF-alpha, interleukins, chemokines, growth factors (TGF-beta, PDGF, IGF-I, hepatocyte growth factor), reactive oxygen species, and vitamin A.

LSECs perform key filtration functions due to small diaphragm-windowed fenestrae or sieve plates that allow free diffusion of many substances between the blood and the hepatocyte surface. LSECs exhibit endocytic capacity for glycoproteins, components of the extracellular matrix (such as hyaluronate, collagen fragments, fibronectin, or chondroitin sulphate proteoglycan), transferrin, ceruloplasmin, and immune complexes. LSECs may also serve as MHC-I- and MHC-II-presenting cells involved in antigen-specific T-cell tolerance. They are also active in the secretion of eicosanoids, nitric oxide, cytokines, endothelin-1, and some extracellular-matrix components.

HSCs in the peri-sinusoidal space of Disse are characterized by the presence of well-branched cytoplasmic processes which contact endothelial cells. In the normal liver, HSCs (i.e. Ito cells) are able to store fat droplets and vitamin A. HSCs also control extracellular matrix turnover and regulate the contractility of sinusoids. Acute damage to hepatocytes activates transformation of quiescent stellate cells into myofibroblast-like cells that play a key role in the development of inflammatory fibrotic response (177–181). HSCs can also swell or contract due to the presence of smooth-muscle actin to regulate sinusoidal blood flow (186). Constriction or relaxation of HSCs from circulating molecules released by neighboring hepatocytes (carbon monoxide and leukotrienes), endothelial cells (endothelin, nitric oxide, and prostaglandins), Kupffer cells (prostaglandins and nitric oxide), and other stellate cells (endothelin and nitric oxide) (187).

Kupffer cells—intra-sinusoidal endocytic and phagocytic tissue macrophages that reside in the liver—are continuously exposed to gut-derived particulate materials and soluble bacterial products. They can produce reactive-oxygen species, nitric oxide, carbon monoxide, eicosanoids, TNF-α, and other cytokines as part of their innate immune-defense function. Kupffer cells can release these inflammatory mediators and ultimately damage the liver during liver dysfunction or when overexposed to bacterial products. Kupffer cells also engulf and clear senescent and damaged erythrocytes and platelets. In the cancer-research setting, Kupffer cells engulf tumor cells very early during metastasis following the injection of tumor cells into the portal vein (188). Liver macrophages modulate immune responses *via* antigen presentation and suppression of T-cell activation by antigen-presenting sinusoidal endothelial cells *via* paracrine actions of IL-10, prostanoids, and TNF-alpha. Kupffer cells can also secrete enzymes and cytokines during liver injury and inflammation that damage hepatocytes and remodel liver tissues.

Pit cells—liver-associated large granular lymphocytes like natural-killer cells—kill a variety of tumor cells in a major histocompatibility complex, unrestricted way, and this anti-tumor activity may γδ T cells, both conventional- and unconventional α- and β-T cells, or liver-sinusoidal natural-killer cells.

Single-cell sequencing technology has revealed more granular levels of cellular heterogeneity (6). These studies identified previously unknown subtypes of endothelial cells, Kupffer cells, and hepatocytes, associated with specific zonal population distributions (see data link: http://human-liver-cell-atlas.ie-freiburg.mpg.de). A zonal division of labor appears to exist within the organ (189, 190). Normal liver-lobule, metabolic, sub-specialization has been divided into the periportal zone that surrounds the portal triad (portal vein, hepatic artery, and bile duct), the central zone closest to the central vein, and the remaining mid zone (189–192). Single-cell sequencing of human liver has also uncovered distinct macrophage populations in those locations (193).

### Tumor Metastasis of the Liver

Ultrastructural studies of experimental metastasis to the liver were first reported by Dingemans (153, 154) (**Figure 5B**), who injected mammary carcinoma cells into syngeneic C57/Bl6 mice. Almost immediately after mesenteric-vein injection, tumor cells had formed large emboli in the portal branches. Tumor emboli adhered to the vascular wall without completely occluding the lumen. On the side of the embolus facing the vascular lumen, aggregated platelet clusters were found as part of the first response in the liver. On the luminal side of the platelet clusters, leukocytes, especially neutrophils, had adhered. Erythrocytes, by contrast, were present near the non-platelet-involved tumor cell surfaces, potentially suggesting that immune cells were preferentially attracted to the aggregated platelets. An outer zone consisting of the platelet aggregates was formed by degranulated- and more or less spherical platelets. The platelet aggregate centers consisted of closely packed, elongated elements along with small amounts of fibrin within heterogenous emboli, which were gradually displaced during metastatic growth and disease progression.

### Liver Coagulation-Factor Biology

Along with regulating platelet number, the liver also plays an important role in coagulation. Both coagulation and anti-coagulant proteins are primarily made in the liver; thus, any liver disease can potentially dysregulate coagulation (156, 194–196). Most coagulation factors are synthesized by the parenchymal cells of the liver (factors I, -II, -V, -VII, -IX, -X; proteins C, -S, and –Z; fibrinogen; antithrombin; α2-PI; and plasminogen); while factor VIII is produced by liver LSECs (197). Of these, the synthesis of factors II, -VII, -IX, -X; and proteins C and -S are dependent on vitamin K—an important cofactor for regulating coagulation. As the primary storage site for vitamin K, the liver provides conversion of synthetic vitamin K to its active form and produces the bile salts that aid with the absorption of food-based vitamin K (156, 194–196, 198). The liver also plays a role in the clearance of the coagulation products from the bloodstream and regulates anticoagulation by removing activated clotting- and fibrinolytic factors *via* the hepatic reticuloendothelial system.

### Liver Damage or Injury and Coagulation

Liver disease can manifest through several mechanisms. Overuse of certain drugs or alcohol, metabolic syndrome, diabetes, chronic viral infection, and exposure to toxins are some of the many contributors to liver injury and disease. Depending on the degree of liver damage, individuals with liver disease have deficiencies in clotting enzymes, which manifest as prolonged clotting times in *in vitro* assays. Chronic liver-disease-associated coagulation disorders result from the inability of the liver to produce or clear clots (194, 199). Imbalances in the synthesis or clearance of clotting factors can increase the risk of bleeding; increasing evidence suggests that these also increase the risk of prothrombotic events (156). The development of coagulopathies is associated with chronic liver disease; circulating levels of some coagulation factors such as vWF and factors II, -V, and -VII have been shown to correlate with the severity of liver disease. Blood coagulation-protein levels can also reflect liver-cell functionality (200–202). In some instances, irregularities in these levels can contribute to the process of liver damage.

#### Sterile Injury

Two types of liver injury—sterile and non-sterile—are mediated by similar but distinctly different platelet engaging immune responses. In the case of sterile injury, an inflammatory reaction occurs in the absence of infection during normal sterile-wound healing (7). When this process becomes dysregulated in various acute- and chronic inflammatory liver diseases (such as non-alcoholic fatty-liver disease [NAFLD]/NASH), toxic injury-altered immune-cell trafficking and abnormal function further promote progressive, chronic inflammatory disease (7). Platelets initiate the early biological responses to injury through direct contact and release of granules (173). In one sterile-injury-and-repair model of the liver, platelets were the first responders directly observed to form a substratum that facilitated neutrophil entry to the injured site for subsequent repair (8). In the sterile-inflammation case of liver ischemia/reperfusion injury, the release of damage-associated molecules can also trigger toll-like receptor 4 (TLR4)- and TLR9-MyD88 signaling pathways to form neutrophil extracellular traps (NETs) that exacerbate organ damage and initiate inflammatory responses (203). Following sterile injury to the liver, local cytokines can also reprogram classic, proinflammatory (CCR2^hi^CX3CR1^low^) monocytes into nonclassical-, patrolling-, or alternative (CCR2^low^CX3CR1^hi^) monocytes to facilitate proper wound-healing (204).

#### Septic Injury

Similar mechanisms are involved in a septic insult to the liver (205, 206). Severe sepsis induces dysregulated inflammation and coagulation leading to multiple organ (particularly liver) failure. Platelet TLR4 receptors initiate the formation of NETs to ensnare pathogens (205, 206). These platelet-initiated NETs and the extravasated platelet aggregation facilitate detachment of LSECs and trigger sepsis-induced liver dysfunction (205, 206). In the case of CRCs, invasive microorganisms like the bacterial species *Fusobacterium nucleatum* are often present (207). These and other bacteria can cause platelet aggregation and have been associated with cancer cells in metastatic lesions (207–209).

#### Liver Fibrosis

Liver disease is often associated with a marked decrease in the synthesis of proteins involved in coagulation. Whereas the levels of some factors like factor VIII, fibrinogen, and vWF remain unchanged or even increase due to defects in their clearance mechanism. In most cases, the defects in liver function are stabilized by between-coagulant- and anti-coagulant-protein counterbalancing. However, irreparable liver damage caused by conditions such as viral hepatitis and fatty liver can result in fibrosis of the liver. Some coagulation proteins—especially the prothrombotic factors—initiate microthrombi formation which could accelerate the progression of fibrosis. Studies in animal models have shown that factor Xa and thrombin contribute to liver fibrosis by occluding hepatic sinusoids with fibrin deposits (thus activating signaling pathways that promote a pro-fibrinogenic phenotype of the liver cells) or by triggering inflammation (156). Liver fibrosis, when severe, can progress into liver cancer and/or other life-threatening conditions. Patients with liver cirrhosis have an increased risk of all-cause mortality due to conditions like increased bleeding and thrombosis that can lead to severe, acutely life-threatening events like pulmonary thromboembolism. Budd-Chiari syndrome, which involves thrombotic occlusion and metastatic-tumor invasion of the hepatic veins, can also cause elevated portal pressures and ascites (210–212). Venous thromboembolism becomes particularly challenging in these patients (213). In others, conditions like liver cirrhosis may lead to a spectrum of coagulation defects, and in rare cases could even progress to disseminated intravascular coagulation—a common feature of end-stage liver disease—with widespread activation of coagulation, fibrinolysis, and hepatic failure. End-stage liver disease is also associated with thrombocytopenia and platelet dysfunction (214).

#### Therapy-Induced

Damage to the liver and hepatotoxicity can result from chemotherapy. This chemotherapy-induced injury is idiosyncratic and can range from asymptomatic, reversible, functional defects to advanced-stage liver disease like cirrhosis. Multiple factors like reactive-oxygen radicals, mitochondrial dysfunction, and immune dysregulation could affect the extent and degree of hepatotoxicity depending on the type of treatment received. Additionally, pre-existing liver conditions including nonalcoholic steatohepatitis are often aggravated by chemotherapy (215–217). Increased levels of activated platelets can also be identified in association with alcohol-induced liver cirrhosis and nonalcoholic steatohepatitis (218–220). Patients with hepatic sinusoidal-obstruction syndrome—associated with oxaliplatin-based chemotherapy (221)–experience portal hypertension, elevated liver enzymes, and splenomegaly, all of which can result in liver atrophy and fibrosis. Development of sinusoidal-obstruction syndrome in patients with CRC-based liver metastasis prior to liver resection surgery can lead to increased morbidity (221, 222). Increased incidence of liver damage is also emerging in association with checkpoint-blockade immunotherapy (223).

Cancer is a hypercoagulable state. Cancer-associated thrombosis increases the risk of morbidity and mortality in cancer patients (224). The risk of venous thromboembolism is approximately 4-fold higher in cancer patients than in normal individuals. Cancer-associated thrombosis also correlates with metastasis. Cancer cells can activate the coagulation cascade *via* signaling mechanisms and secretion of cytokines. Moreover, tumor burden can cause vessel compression eventually leading to thrombosis (224). Platelets are primary contributors in coagulation and thrombogenesis pathways. Platelet counts and activation markers have a significant impact on the prognosis of cancer and response to therapy (4, 225, 226). Intriguingly, cancer treatment, including surgery, also has an impact on platelet activity, which could increase hypercoagulability in these patients. In one study of cancer patients receiving chemotherapy, the risk of thrombosis was increased by 6- to 7-fold (227). Chemotherapy may also contribute to an increased risk of thrombotic events and venous thromboembolism, both associated with decreased survival. In another study, oxaliplatin-based chemotherapy affected platelets in the liver; the number attached to liver cells positively correlated with sinusoidal-obstruction syndrome severity (221). Oxaliplatin-based damage to hepatic sinusoids can possibly attract and activate platelets (228–230). Once activated, platelets secrete growth factors such as platelet-activating factor and thromboxane A_2_, causing liver injury, vascular and sinusoidal occlusion, and collagen deposition (231, 232). Then again, high-dose bevacizumab may interfere with platelet activation (228–230). The increased risk of bleeding in patients receiving bevacizumab treatment could be attributed to down-regulation of platelet activation (229). The impact of systemic cancer therapy is likely to vary along with the patient’s cancer type, performance status, comorbidities, individual platelet biology, and type of treatment (233–238).

## Liver Disease and Cancer Risk

Liver disease contributes to an increased risk of CRC through the gut-liver axis (239). As the liver plays important roles in metabolism, synthesis and regulation of hormones, microbiome factor clearance, and blood detoxification, any major alterations in the liver function can lead to clinical findings including hypertension, diabetes, and hyperlipidemia. Systemic alterations stemming from liver dysfunction also can lead to dysregulated cytokine production and immune-cell function that initiate malignant transformation of cells and/or promote survival of cancer cells in the liver. Fatty liver is a known risk factor for CRC and cirrhosis patients have nearly double the risk of developing the CRC (240). CRC is known to be associated with thrombocytosis, hypercoagulation, and thromboembolic events (241, 242). About 35-55% of CRC patients develop liver metastasis, and surgical resection of the metastasis can be curative in some of these patients (243). In most cases, systemic chemotherapy is used prior to and after resection. This becomes challenging in patients with pre-existing- or with chemotherapy-induced liver disease as the goal of liver-resection surgery is to preserve liver functionality by removing metastasis. Patients with liver cirrhosis have a higher mortality rate following CRC surgery (244).

As the liver is actively involved in the synthesis and regulation of hormones, chronic hepatocellular damage can lead to hormonal imbalance. Estrogen levels are elevated in men with alcoholic liver disease and in others with a high body-mass index. Liver cirrhosis in men increases their risk for breast cancer possibly due to hyperestrogenemia (245–247). Steroid imbalance is also associated with viral cirrhosis and hepatocellular carcinoma (248). Evidence suggests that irrespective of cirrhosis type, all patients with cirrhosis and any related metabolic changes have an increased risk for liver cancer (249, 250). Studies also show that NAFLD is positively correlated with pancreatic cancer, and that NAFLD may serve as a prognostic factor; patients with pancreatic cancer and NAFLD have poorer overall survival than those without NAFLD (251). In addition, coagulation-factor Xa was shown to promote tumor growth and metastasis in animal models of melanoma (166).

### Platelet Activation and Aggregation

Trousseau first reported excessive blood coagulation in cancer patients with elevated platelet counts or thrombocytosis in 1865 (252–254). Since then, numerous studies have reported on thrombocytosis in cancer. In ovarian cancer, thrombocytosis is linked to elevated tumor interleukin-6 and liver-generated thrombopoietin and is associated with shortened overall survival of patients (255). In one study, orthotopic ovarian-mouse models revealed tumor-derived, interleukin-6-stimulated, hepatic-thrombopoietin (TPO) synthesis and paraneoplastic induction of thrombocytosis (255). Liver metastasis along with Fusobacterium in the liver can also trigger the thrombotic Trousseau’s syndrome (256–258). Observed abnormalities associated with coagulation factors like fibrinogen and prothrombin in liver disease could activate platelets (259). In certain conditions, platelets interact and bind with endothelial cells, hepatocytes, hematopoietic stem cells, and Kupffer cells in the liver (173). Upon injury to the liver endothelium, platelets are sequestered within the sinusoid and lead to endothelial-cell activation. This activation releases cytokines that facilitate the infiltration of immune cells (175). Recruitment of neutrophils and macrophages by platelets is of importance in liver disease as they regulate inflammation, fibrinogenesis, and fibrinolysis, and contribute to fibrosis (260). Platelet-mediated neutrophil recruitment and the interaction of platelets with the vasculature have been suggested as mechanisms driving cholestasis-induced liver damage (261). In NAFLD, the severity of inflammation and fibrosis in the liver was shown to directly correlate to the increased platelet turnover (262). In cirrhosis, the elevated levels of vWF promote platelet binding to collagen (263). Damage to the liver can directly affect liver-produced TPO levels. Circulating TPO levels have been shown to negatively correlate with the stage of liver disease, while reduced levels can lead to decreased platelet production or thrombocytopenia. In addition, thrombocytopenia in chronic liver disease is also caused by hypersplenism and increased sequestration of splenic platelets and increased platelet destruction due to aggregation in the liver (264). Increased thrombosis in these patients may consume platelets leading to lower levels in circulation (265). PDGF‐β, a mediator of hepatic fibrosis, is produced by platelets and released upon their activation (266). With the established association between platelet activation and liver fibrosis, anti-platelet drug use could prove beneficial in combating platelet-mediated liver disease and progression to cirrhosis. Recently, a systemic review and meta-analysis report of 3,141 patients concluded that the use of anti-platelet agents (such as aspirin or clopidogrel) was associated with a 32% decreased odds of hepatic fibrosis (adjusted pooled OR 0.68; CI 0.56–0.82, p ≤ 0.0001) (267). However, a prospective cohort study of patients at high risk of cardiovascular events revealed an inverse association between the use of anti-platelet agents and the presence and degree of liver fibrosis (268). A recent study of patients with hepatocellular carcinoma demonstrated that anti-platelet therapy was associated with improvement in overall survival and reduction in liver-related deaths. This study also reported that the use of anti-platelet therapy tended to delay the deterioration of liver function in these patients (269). Recent summaries of outcomes data in cancer prevention have also highlighted the cancer mitigating role of aspirin and other agents (270).

#### Tumor-Educated Platelets and Exosomes

Tumor-derived exosomes—vesicles secreted by tumor cells—are packed with proteins and nucleic acid content that differs among different tumor types (271). These cancer exosomes may prime the liver environment to influence metastasis and can influence diverse cell types ranging from immune cells, endothelial cells, and platelets. Tissue factor—involved in the initiation of clot formation—may be enriched in cancer exosomes which may trigger increased thrombosis (272). Studies have also shown that microvesicles can bind and fuse with activated platelets to initiate coagulation (273).

Platelets also release a variety of vesicles upon activation, ranging from smaller ectosomes to larger exosomes. Platelet microvesicles make up the bulk of circulating microparticles in the blood stream (271, 274–278). These vesicles carry mRNA, miRNA, proteins, and lipids and are known to be critical in the process of angiogenesis, cancer progression, and metastasis (279, 280). When cells in the vicinity of platelet activation come into contact with the cargo in these vesicles, gene-based modulation of protein expression in target cells may occur (281). These vesicles can fuse with tumor cells and transfer receptors inducing chemotaxis, expression of metalloproteases, and cell proliferation. These exchanges may contribute to the development of drug resistance in tumor cells (282, 283). Recent findings provide insight into horizontal-RNA transfer mechanisms between platelets, platelet microparticles, and tumor cells (271, 284). Tumor-educated platelets appear to undergo modifications when they come into contact with tumor cells. These platelets may retain tumor-specific information including the primary tumor location (285, 286).

### Platelet Activation and Liver Metastasis

The liver is a common site for cancer metastasis from primary tumors originating in the gastrointestinal tract. Traditionally the preponderance of liver metastasis was felt to be a function of drainage from the intestines into the hepatic circulation; however, the liver may instead represent a favorable microenvironment for metastasis. The liver’s unique regeneration may likewise contribute to creating a receptive metastasis-formation microenvironment involving platelets and the chronic activation of pathways related to the Myc family of regulator genes (287–292). More than half of CRC patients develop liver metastases (associated with poor prognosis) (85, 225) and an increased risk of thrombosis (241, 293). A positive correlation has been observed between high platelet counts and CRC tumor invasiveness and -metastasis, and a negative correlation between high platelet counts and survival in CRC patients (294–296). Platelet hyperactivation measured by elevated platelet mean volume or aggregation (297–301) can be a predictor of cancer progression in CRC (302–304). Genes implicated in CRC are also involved in platelet activation and coagulation suggesting a prothrombotic environment in CRC (305). During cancer progression and metastasis, platelet responses can be modulated by the tumor cells. Circulating tumor cells (CTCs) can stimulate heterotypic tumor-cell-induced platelet aggregation (TCIPA) (29, 85, 306). Tumor cells may get trapped within a TCIPA, go undetected by immune surveillance, be protected from shear forces while in circulation, and have greater capacity to migrate and metastasize (61, 271). Recently, a microfluidic approach was developed to isolate CTCs by targeting platelets that satellite on the tumor-cell surfaces. A significant number of platelet-coated CTCs was observed in metastatic cancer patients of both epithelial (lung and breast)- and non-epithelial (melanoma) tumor origins. Also observed were single cells and clusters, along with CTCs associated with leukocytes (307). Isolating CTCs using platelet markers emphasizes the potential of platelet-coated CTCs that go unnoticed by conventional isolation methods and their possible significance in metastasis.

Tumor cells also release thrombin which serves to activate platelets and results in the formation of tumor thrombi, a form of TCIPA. These thrombi could provide a supportive environment for CTCs by becoming tethered to blood vessels within distant tissues. Given the heightened coagulatory imbalances in the liver driven by cancer or pre-existing- and/or chemotherapy-induced chronic liver disease, these thrombi can become lodged within the liver sinusoids, potentially leading to the initiation of metastasis. Many studies have used anti-platelet drugs with chemotherapy to reduce platelet-mediated tumor-cell survival and metastasis. Platelets were also shown to promote angiogenesis by releasing angiogenic growth factors such as VEGF (308). In a study that provided aspirin along with tamoxifen therapy to patients with breast cancer investigated the impact of aspirin therapy on circulating levels of the proangiogenic-protein VEGF, the antiangiogenic-protein TSP‐1, and platelet-mediated angiogenic-protein release. The aspirin therapy not only impacted angiogenic protein levels but it may modify the angiogenic balance in women treated with tamoxifen therapy. The increase in antiangiogenic TSP‐1 levels without a concurrent increase in pro‐angiogenic VEGF levels suggests an anti-angiogenic balance from aspirin therapy. This study also found less release of platelet angiogenic proteins (309).

In addition to the pathways discussed previously, dysregulated modulation of TGFβ activity can also provide a favorable environment for tumorigenesis (76). Platelets are the major source of latent TGFβ which is released during platelet activation. This process also releases a furin-like proprotein convertase from platelets which in turn activates TGFβ. This enzyme-mediated activation was shown to continue in the damaged area even after platelet-derived TGFβ activation was complete (75). Aberrant expression of TGFβ could lead to liver fibrosis. Elevated levels of vWF can increase platelet binding to collagen in the cirrhotic condition (263). Thus, conditions like liver cirrhosis can increase the activation and aggregation of platelets, with TGFβ and other growth-factor release not only causing platelet binding with collagen, but also providing a tumorigenic niche within these platelet-collagen traps to capture CTCs and promote their growth within the liver.

### Platelets and Minimal Residual Disease

Minimal residual disease (MRD)—a collection of viable cancer cells that are undetectable by standard imaging methods—differs by tumor type and requires disease-specific stratification based on distinctive organ-related microenvironmental disease characteristics (310). Recent discoveries in the development and use of liquid biopsies based on analyses of biomarkers in body fluids—including blood, urine, and cerebrospinal fluid—are showing potential for use in stratifying MRD. Liquid biopsy analyses can include (1) tumor-derived DNA, -RNA, -miRNA; epigenetic alterations; and proteins present in cell-free plasma or (2) contained in CTCs or (3) circulating exosomes and microvesicles or tumor educated platelets (271, 285, 286, 311–314). To fully understand MRD from a liquid biopsy standpoint, the inclusion of proteomics and metabolomics may be desirable to form a complete integrated molecular profile. To help with this, animal models of MRD involving gut related vascular modes of injection are materializing, which can help represent MRD in CRC (315, 316). Exposing platelets to tissue-factor-expressing tumor cells in the MRD microenvironment, may also trigger the activation cascade that promotes disease progression.

Based on our experience in developing in-house assays for solid tumor ctDNA (317), the detection of blood-based circulating-tumor DNA (ctDNA), a prognostic biomarker highly sensitive for CRC recurrence following curative-intent therapy, identifies MRD that will inevitably develop into clinically detectable, local recurrent- and/or distant metastases (318–321). Currently, ctDNA assays that detect MRD are also commercially available as a standard-of-care tool that enables oncologists to monitor for CRC recurrence. However, when ctDNA is detected, no prospective data directly linked to proven therapies are available to guide clinical management of CRC (322). A clinical need exists to better understand the biology of micrometastases and the role of platelets, especially in microscopic disease. If platelet-based biomarkers (e.g., platelet-to-lymphocyte ratios or micrometastasis-educated platelets) can portray ctDNA-defined MRD before the onslaught of macroscopic, clinically evident metastatic disease, our diagnosis and window for treating microscopic disease will likely improve before the tumor growth outpaces the immune-system ability to eliminate the disease. Presently, little is known from patient samples about the impact of platelets on the biology of micrometastases, while few therapeutics that consider this potential platelet involvement have been tested in patients.

## Conclusion

Platelets are immediate responders to an injury and contribute to the coagulation process by physically plugging the wound and releasing factors that contribute in the repair process. In addition to platelets, the liver also plays a critical role in hemostasis by synthesizing coagulation pathway proteins and promoting platelet production. In conditions where the liver has sustained significant damage, coagulation- and thrombogenesis processes can become dysregulated. When combined with liver disease, thrombocytosis and platelet activation is further promoted creating a microenvironment similar to that of a wound. Activated platelet-surface interactions and granule release initiate signal transduction and biologic responses.

Analogous mechanisms come into play in the case of cancer. Platelet activation, circulating cancer exosomes, thrombosis, TCIPA, and heterotypic aggregate formation are commonly associated with cancer. In patients with cancer and liver disease the interactions between CTCs and activated platelet aggregates can increase, and platelet-tumor thrombi can get trapped within the hypercoagulative liver vasculature. The administration of chemotherapy can further worsen the hemostatic regulation in these patients. The increased formation of Trousseau’s-Syndrome-related thrombi and the increased interaction of platelets together with tumor cells create a suitable environment for tumor-cell entrapment. Given the complex, multi-faceted role of the platelet in both wound healing and cancer biology, it remains uncertain if certain cancer patients could benefit from platelet-inhibition agents not yet identified.

In conclusion, heightened liver injury may increase the retention of CTCs that triggers rapidly responding platelets and fibrinogenesis and associated immune cells at metastatic foci within liver sinusoids. In patients who have a pre-existing liver disease or who develop chemotherapy-induced liver injury, these CTCs are likely to find a supportive niche within the liver sinusoids and adjacent space of Disse that promotes liver metastasis and MRD.

## Author Contributions

PK and NF contributed equally. All authors contributed to the article and approved the submitted version.

## Funding

DM is supported by Institutional Research Grant from UT MD Anderson Cancer Center. SK, DM, PKM, AD, JS, NY, and MO are supported by the Colorectal Cancer Moon Shot. SK, DM, JS and MO are supported by 2P30CA016672-43, 1P50CA221707-01A1, 5R01CA218230-02 and 5R01CA184843-05. AKS is supported by P50 CA217685, R35 CA209904, the American Cancer Society, and the Frank McGraw Memorial Chair in Cancer Research. PK is supported by Cancer Prevention Research Training Program, the Janice Davis Gordon Memorial Postdoctoral Fellowship in Colorectal Cancer Prevention Research, University of Texas MD Anderson Cancer Center. VAK is supported by the National Cancer Institute (CA231141, CA177909) and McCullough Professorship for Cancer Research. JS is supported by the Cancer Prevention & Research Institute of Texas as a CPRIT Scholar in Cancer Research (RR180035) and the National Cancer Institute (L30 CA171000 and K22 CA234406).

## Conflict of Interest

AKS: Consulting (Merck, Kiyatec), shareholder (BioPath), research funding (M-Trap).

The remaining authors declare that the research was conducted in the absence of any commercial or financial relationships that could be construed as a potential conflict of interest.

## Abbreviations

CTCs, Circulating tumor cells; CRC, colorectal cancer; EGF, epidermal growth factor; HSCs, hepatic stellate cells; LSECs, liver sinusoidal endothelial cells; MRD, minimal residual disease; NAFLD, non-alcoholic fatty liver disease; NASH, non-alcoholic steatohepatitis; PDGF, platelet-derived growth factor; TPO, thrombopoietin; TLRs, toll-like receptors; TCIPA, tumor-cell-induced platelet aggregation; VEGF, vascular endothelial growth factor; vWF, vonWillebrand factor.
